# Short-term prognosis of low-risk prostate cancer patients is favorable despite the presence of pathological prognostic factors: a retrospective study

**DOI:** 10.1186/s12894-023-01345-z

**Published:** 2023-10-31

**Authors:** Remi Semba, Katsunori Uchida, Yoshihumi Hirokawa, Taizo Shiraishi, Takehisa Onishi, Takeshi Sasaki, Takahiro Inoue, Masatoshi Watanabe

**Affiliations:** 1 Department of Oncologic Pathology, Mie University Graduate School of Medicine, 2-174 Edobashi, Tsu, Mie 514-8507 Japan; 2 Department of Pathology, Kuwana City Medical Center, Kuwana, Mie Japan; 3 Department of Urology, Japanese Red Cross Ise Hospital, Ise, Mie Japan; 4 Department of Nephro-Urologic Surgery and Andrology, Mie University Hospital, Tsu, Mie Japan

**Keywords:** Biochemical recurrence-free survival, Prostate cancer

## Abstract

**Background:**

Prostate cancer patients with pathological prognostic factors have a poor prognosis, but it is unclear whether pathological prognostic factors are associated with prognosis limited to low-risk patients with good prognosis according to NCCN guidelines. The present study examined whether prognosis is influenced by pathological prognostic factors using radical prostatectomy (RP) specimens from low-risk patients.

**Methods:**

We evaluated diagnostic accuracy by examining biochemical recurrence (BCR)-free survival with respect to clinical and pathological prognostic factors in 419 all-risk patients who underwent RP. Clinical prognostic factors included age, prostate-specific antigen (PSA) levels, PSA density, and risk stratification, while pathological prognostic factors included grade group, lymphovascular space invasion, extraprostatic extension, surgical margins, seminal vesicle invasion, intraductal carcinoma of the prostate (IDCP), and pT. In a subsequent analysis restricted to 104 low-risk patients, survival curves were estimated for pathological prognostic factors using the Kaplan–Meier method and compared using log-rank and generalized Wilcoxon tests.

**Results:**

In the overall risk analysis, the presence of pathological prognostic factors significantly shortened BCR-free survival (*p* < 0.05). Univariable analysis revealed that PSA density, risk categories, and pathological prognostic factors were significantly associated with BCR-free survival, although age and PSA were not. In multivariable analysis, age, risk categories, grade group, IDCP, and pT significantly predicted BCR-free survival (*p* < 0.05). Conversely, no statistically significant differences were found for any pathological prognostic factors in low-risk patients.

**Conclusions:**

In low-risk patients, pathological prognostic factors did not affect BCR-free survival, which suggests that additional treatment may be unnecessary even if pathological prognostic factors are observed in low-risk patients with RP.

## Background

Prostate cancer (PCa) is a clinically heterogeneous disease, ranging from non-life-threatening “insignificant cancers” to poor-prognosis cancers that metastasize and cause death. Therefore, pre-treatment risk stratification is important to determine the appropriate treatment. The NCCN Clinical Practice Guidelines in Oncology (NCCN guidelines) classify PCa into very low-, low-, favorable/unfavorable intermediate-, high-, and very high-risk categories based on clinical stage, grade group (GG), prostate-specific antigen (PSA) levels, PSA density (PSAD), number of positive cores at biopsy, and occupancy rates [1]. A decision tree for treatment options is organized according to expected patient survival and risk category. For very low- and low-risk patients, treatment options include active surveillance (AS), external beam radiotherapy (EBRT), brachytherapy　(BT), or radical prostatectomy (RP).

Prognostic factors for biochemical recurrence (BCR) of clinically or pathologically localized PCa include clinical prognostic factors such as age [2], PSA [3], and PSAD [4], and pathological prognostic factors such as GG [5], lymphovascular invasion (LVI) [6], extraprostatic extensions (EPE) [7], surgical margins (SM) [8], seminal vesicle invasion (SVI) [9], and pT [10]. Additionally, intraductal carcinoma of the prostate (IDCP) has recently been considered an independent and strong prognostic factor [11]. IDCP is morphologically similar to the cribriform pattern, but IDCP proliferates within pre-existing ducts. Both IDCP and cribriform pattern are poor prognostic factors, but the distinction is sometimes difficult. The presence of these pathological prognostic factors results in a worse prognosis, even in the short-term.

Low-risk patients have significantly higher BCR-free survival rates than intermediate- and high-risk patients [12], but existing studies of low-risk patients with RP showed that EPE was found in 8–36%, SVI in 1.5%, and positive SM in 19–33% of RP specimens [13–15]. The prevalence of IDCP in low-risk patients has been reported to be 2.1% [16]. These treatises did not examine whether pathological prognostic factors in low-risk patients were associated with prognosis. Thus, it remains unclear whether the presence of pathological prognostic factors in low-risk patients who are considered to have a good prognosis is associated with a poor prognosis. Here, we report the results of a study attempting to elucidate this.

## Materials and methods

### Patient selection

**Fig. 1:**
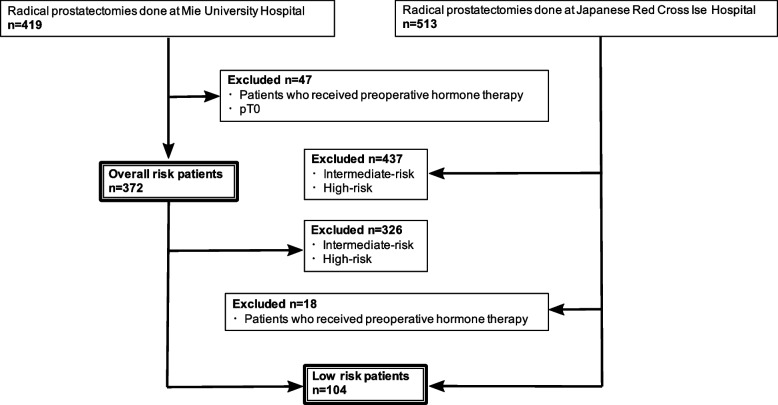
The flow chart of patient selection

A flowchart of patient selection is shown in Fig. 1. We performed a retrospective review of data collected from the database which included 419 patients that underwent RP at Mie University Hospital between 2015 and 2021. Of the 419 patients identified, 46 patients who received preoperative treatment and 1 whose cancer was not detected postoperatively were excluded, leaving 372 patients in the overall risk analysis.

For the analysis of the low-risk group, patients from the Japanese Red Cross Ise Hospital were included to increase the sample size for statistical analysis. Of the 513 patients who underwent RP at the Japanese Red Cross Ise Hospital between 2012 and 2021, 76 low-risk patients were enrolled in our study after excluding those who received preoperative hormone therapy. There were no very low-risk patients among those who underwent RP at the above two hospitals.

Patients were treated according to the NCCN guidelines. For low-risk patients, treatment options included AS, EBRT, BT, or RP. Patients could choose the treatment of their choice. This study, in collaboration with the Japanese Red Cross Ise Hospital, was approved by the Mie University Clinical Research Review Committee (approval number H2020-110), and the requirement for obtaining informed consent from the participants was waived owing to the retrospective and observational nature of the study.

### Medical record data and pathological evaluation

Preoperative clinical prognostic variables (age, PSA, PSAD) and biopsy results were retrieved from patient records. Risk classification was based on the NCCN guidelines (version 4, 2022) [1]. Low-risk patients were defined as cT1 ~ cT2a and GG1 and PSA < 10 ng/mL.

RP specimens were fixed in 10% neutral buffered formalin, and hematoxylin and eosin-stained (HE-stained) slides were prepared. The pathological prognostic factors in RP specimens for BCR, GG, LVI, EPE, SM, SVI, IDCP, and pT were independently re-evaluated by two experienced genitourinary pathologists (RS and KU). GG and IDCP were evaluated according to the 2016 WHO classification [17].　We diagnosed IDCP with only HE-stained slides and did not use immunohistochemistry. EPE is diagnosed when cancer bulges beyond the boundaries of the prostate and does not include bladder neck invasion. SM was defined as positive if cancer cells were in contact with the specimen surface. pT was evaluated based on the TNM Classification of Malignant Tumors, 8th edition [18].

### Follow up

Serum PSA was measured after RP every 3 months for the first 3 years and every 6 months thereafter. Imaging studies (including computed tomography and bone scintigraphy) were performed as needed.

BCR was defined as a rise in PSA to 0.2 ng/mL and a confirmatory value of 0.2 ng/ml. The first PSA above or equal to 0.2 ng/ml was used to define the time to BCR. Even if EPE, positive SM, or SVI were observed in RP specimens, no additional treatment was given until BCR was confirmed.

### Statistical analyses

The primary endpoint of this study was BCR-free survival, defined as the time from RP to BCR. For the overall risk analysis, survival curves were estimated for risk categories and pathological prognostic factors using the Kaplan–Meier method and compared using log-rank and generalized Wilcoxon tests. For clinical and pathological prognostic factors, Cox proportional hazards regression models were used to estimate relative risks and 95% confidence intervals for unadjusted and adjusted comparisons of BCR. Univariable and multivariable Cox proportional hazards models were built with BCR-free survival as the outcome and clinical and pathological prognostic factors as the covariates. The multivariable model was adjusted for age, risk categories, GG, IDCP, and pT. PSA and PSAD were excluded from the multivariable analysis because they were associated with risk classification, and EPE, SM [19], and SVI were also excluded because of their association with pT.

For the analysis of low-risk patients, survival curves were estimated for pathological prognostic factors using the Kaplan–Meier method and compared using log-rank and generalized Wilcoxon tests. Nominal variables were calculated using Fisher's exact test, and continuous variables were calculated using the *t*-test and Mann–Whitney U test.

*P* value < 0.05 was considered statistically significant. All statistical analyses were performed using EZR version 1.54 (Saitama Medical Center, Jichi Medical University, Saitama, Japan), which is a graphical user interface for R version 4.1.1 (The R Foundation for Statistical Computing, Vienna, Austria).

## Results

### Clinical characteristics of the study participants

The clinicopathological characteristics of the 372 patients included in the overall risk analysis are summarized in Table 1. The median age of patients was 68 years (range: 43–76 years), median PSA was 7.8 ng/ml (range: 2.78–90.5 ng/ml), and median PSAD was 0.3 ng/mL/cc (range: 0.06–5.36 ng/mL/cc). Risk stratification of patients based on the NCCN guidelines was as follows: low-risk, 12% (*n* = 46); favorable intermediate-risk, 26% (*n* = 96); unfavorable intermediate-risk, 22% (*n* = 80); high-risk, 34% (*n* = 127); and very high-risk, 6% (*n* = 23). The GGs based on the 2016 WHO classification in patients were GG1 in 41(11%), GG2 in 141 (38%), GG3 in 90 (24%), GG4 in 36 (10%), and GG5 in 64 (17%). In addition, LVI, EPE, positive SM, SVI, and IDCP were found in 37 (10%), 105 (28%), 111 (30%), 30 (8%), and 66 (18%) patients, respectively. pT was classified as pT2 in 265 (71%), pT3a in 77 (21%), and pT3b in 30 (8%) patients. The median follow-up duration was 18 months (range: 0–74 months), and no patient died of PCa during follow-up. Forty-seven patients (13%) had BCR, and the median time to BCR was 16 months (range: 0–72 months).

**Table 1:** Clinical and pathological characteristics of patients included in the overall risk analysis (*n* = 372)

Overall Risk Patients Analysis (*n* = 372)			
Age (yr)	Median (range)		68 (43–76)
PSA	Median (range)		7.8 (2.78–90.5)
PSAD	Median (range)		0.3 (0.06–5.36)
Risk Stratification	Low (%)		46 (12)
	Intermediate	Favorable (%)	96 (26)
		Unfavorable (%)	80 (22)
	High (%)		127 (34)
	Very high (%)		23 (6)
GG^a^	1 (%)		41 (11)
	2 (%)		141 (38)
	3 (%)		90 (24)
	4 (%)		36 (10)
	5 (%)		64 (17)
LVI	Positive (%)		37 (10)
EPE	Positive (%)		105 (28)
SM	Positive (%)		111 (30)
SVI	Positive (%)		30 (8)
IDCP	Positive (%)		66 (18)
pT	2 (%)		265 (71)
	3a (%)		77 (21)
	3b (%)		30 (8)
Follow-up Duration (mo)	Median (range)		18 (0–74)

*PSA* Prostate-specific antigen, *PSAD* PSA density, *GG* Grade group, *LVI* Lymphovascular invasion, *EPE* Extraprostatic extension, *SM* Surgical margins, *SVI* Seminal vesicle invasion, *IDCP* Intraductal carcinoma of the prostate, *pT* Pathological T stage

^a^GG is in RP specimen

### Overall risk analysis of the study participants

**Fig. 2:**
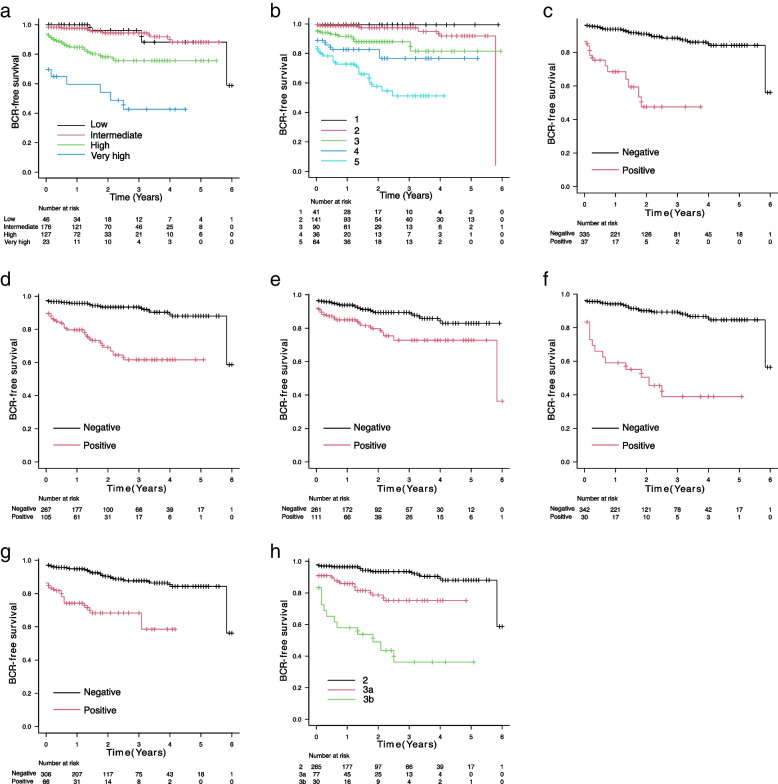
Biochemical recurrence-free survival for the NCCN risk stratification and pathological prognostic factors in all patients. Kaplan–Meier estimates of biochemical recurrence (BCR)-free survival for all risk category patients according to the NCCN risk stratification (**a**), grade group (GG) (**b**), lymphovascular invasion (LVI) (**c**), extraprostatic extension (EPE) (**d**), surgical margins (SM) (**e**), seminal vesicle invasion (SVI) (**f**), intraductal carcinoma of the prostate (IDCP) (**g**), and pathological assessment of the primary tumor (pT) (**h**). The log-rank and generalized Wilcoxon tests showed statistically significant differences for all factors examined (log-rank test: SM *p* = 0.00398, the other factors *p* < 0.001, generalized Wilcoxon test: SM *p* = 0.00355, the other factors *p* < 0.001)

Kaplan–Meier analyses of BCR-free survival stratified according to risk categories, and pathological prognostic factors are shown in Fig. 2a–h. The log-rank and generalized Wilcoxon tests showed statistically significant differences for all factors examined (log-rank test: SM *p* = 0.00398, the other factors *p* < 0.001, generalized Wilcoxon test: SM *p* = 0.00355, the other factors *p* < 0.001). Univariable analysis revealed that risk category, GG, EPE, positive SM, SVI, IDCP, and pT were all significantly associated with BCR-free survival but not age and PSA (Table 2). In multivariable analysis, age, risk categories, GG, IDCP, and pT significantly predicted BCR-free survival (Table 2).

**Table 2:** Univariable and multivariable Cox regression models for biochemical recurrence (BCR)-free survival (*n* = 372)

	**Univariable analysis**		**Multivariable analysis**	
	Hazard ratio (95% CI)	*p*-value	Hazard ratio (95% CI)	*p*-value
Age (year)	1.04 (0.9864–1.096)	0.147	1.058 (1.002–1.116)	0.04211
PSA	1.021 (0.998–1.044)	0.07484	N/A	
PSAD	1.488 (1.05–2.107)	0.02533	N/A	
Risk Stratification	2.11 (1.616–2.754)	< 0.001	1.351 (1.001–1.823)	0.0494
GG^a^	2.365 (1.833–3.05)	< 0.001	1.694 (1.260–2.278)	< 0.001
EPE	4.945 (2.711–9.018)	< 0.001	N/A	
SM	2.258 (1.271–4.013)	0.005499	N/A	
SVI	6.608 (3.6–12.13)	< 0.001	N/A	
IDCP	4.1 (2.267–7.415)	< 0.001	1.929 (1.046–3.557)	0.03539
pT	3.232 (2.287–4.566)	< 0.001	1.972 (1.337–2.908)	< 0.001

*CI* Confidence interval, *PSA* Prostate-specific antigen, *PSAD* PSA density, *GG* Grade group, *LVI* Lymphovascular invasion, *EPE* Extraprostatic extension, *SM* Surgical margins, *SVI* Seminal vesicle invasion, *IDCP* Intraductal carcinoma of the prostate, *pT* Pathological T stage, *N/A* Not available

^a^GG is in RP specimen

### Clinical characteristics of the low-risk patients

The total number of low-risk patients included in the study was 104; 46 patients from Mie University Hospital and 58 patients from the Japanese Red Cross Ise Hospital. The clinicopathological characteristics of 104 low-risk patients are summarized in Table 3. The median age of the patients was 67.5 years (range: 55–77 years), the median PSA was 5.8 ng/ml (range: 2.78–9.7 ng/ml), and the median PSAD value was 0.16 ng/mL/cc (range: 0.06–0.62 ng/mL/cc). The GGs of low-risk patients were GG1 in 25 (24%), GG2 in 59 (57%), GG3 in 16 (15%), GG4 in 3 (3%), and GG5 in 1 (1%). LVI, EPE, positive SM, and IDCP were found in 11 (11%), 13 (13%), 27 (26%), and 10 (10%) patients, respectively. No patient had SVI. pT was pT2 in 89 (86%) and pT3a in 13 (13%) patients. The median follow-up duration was 35.5 months (range: 0–109 months). During follow-up, no patient died of PCa, 6 patients (6%) had BCR, and the median time to BCR was 19.5 months (range: 0–70 months).

**Table 3:** Clinical and pathological characteristics of the low-risk patients (*n* = 104)

Low-Risk Patients Analysis (*n* = 104)		
Age (yr)	Median (range)	67.5 (55–77)
PSA	Median (range)	5.8 (2.78–9.7)
PSAD	Median (range)	0.16 (0.06–0.62)
GG^a^	1 (%)	25 (24)
	2 (%)	59 (57)
	3 (%)	16 (15)
	4 (%)	3 (3)
	5 (%)	1 (1)
LVI	Positive (%)	11 (11)
EPE	Positive (%)	13 (13)
SM	Positive (%)	27 (26)
SVI	Positive (%)	0 (0)
IDCP	Positive (%)	10 (10)
pT	2 (%)	91 (88)
	3a (%)	13 (13)
Follow-up duration (mo)	Median (range)	35.5 (0–109)

*PSA* Prostate-specific antigen, *PSAD* PSA density, *GG* Grade group, *LVI* Lymphovascular invasion, *EPE* Extraprostatic extension, *SM* Surgical margins, *SVI* Seminal vesicle invasion, *IDCP* Intraductal carcinoma of the prostate, *pT* Pathological T stage

^a^GG is in RP specimen

### Overall risk analysis of the low-risk patients

**Fig. 3:**
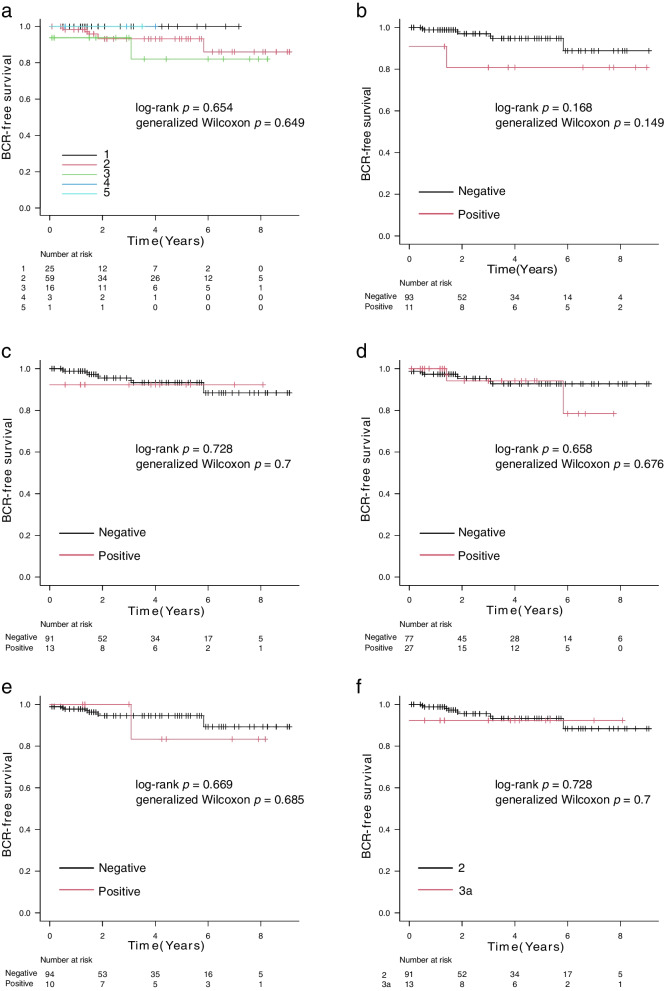
Biochemical recurrence-free survival for pathological prognostic factors in low-risk patients. Kaplan–Meier estimates of biochemical recurrence (BCR)-free survival for low-risk patients according to grade group (GG) (**a**), lymphovascular invasion (LVI) **(b)**, extraprostatic extension (EPE) **(c)**, surgical margins (SM) **(d)**, intraductal carcinoma of the prostate (IDCP) (**e**), and pathological assessment of the primary tumor (pT) **(f)**

Kaplan–Meier analyses of BCR-free survival stratified according to pathological prognostic factors are shown in Fig. 3a–f. The log-rank and generalized Wilcoxon tests did not reveal statistically significant differences for any of the factors examined.

## Discussion

With the widespread use of PSA screening tests, the incidence rates of prostate cancers have increased, and mortality rates have decreased, while the overdiagnosis and overtreatment of clinically insignificant cancers have also been controversial [20]. Given the heterogeneity of PCa, various risk stratifications and nomograms for the pretreatment of PCa have been created. The NCCN, EAU, and other guidelines classify pretreatment PCa into 3–5 risk categories (very low to very high) [21].　Low-risk patients have significantly lower BCR rates than intermediate-risk and high-risk patients [12]. In this study, we examined BCR-free survival for risk stratification only in patients who underwent RP. The overall difference was statistically significant, supporting the validity of our preoperative diagnoses.

Prognostic factors for BCR of clinically or pathologically localized PCa are as follows: clinical factors, including age [2], PSA [3], and PSAD [4], and pathological factors, including GG [5], LVI[6], EPE [7], SM [8], SVI [9], IDCP [11], and pT [10]. In the overall risk analysis of the study participants, the presence of pathological prognostic factors significantly shortened BCR-free survival, as reported in a previous report. In addition, the hazard ratios for positive SM, SVI, and IDCP were higher in the previous report [22], and similarly in our study, the hazard ratios for pT, reflecting positive SM, SVI, and IDCP, were also higher. These results prove that our postoperative pathological diagnosis is appropriate.

Among the pathological prognostic factors, EPE, positive SM, and SVI are defined as adverse pathologic features according to the NCCN guidelines and influence treatment selection [1]. It is not uncommon to find these features in RP specimens from low-risk patients, which are reported to be 8–36% for EPE, 1.5% for SVI, and 19–33% for positive SM [13–15]. In the case of adverse pathologic features, the NCCN guidelines suggest that additional treatment with EBRT or androgen deprivation therapy is an option, regardless of the preoperative risk categories [1]. Though this additional treatment decreases the risk of BCR [23–25], it is reported to decrease urinary function, sexual function, and overall quality of life [26] leading to overtreatment. There is no clear criteria on whether this additional treatment should be administered or followed up. To the best of our knowledge, there are no reports examining the correlation between preoperative risk stratification and additional treatment. In this study, we also examined BCR-free survival for each of the pathological prognostic factors in low-risk patients and found no significant difference in any of the factors, which suggests that additional treatment might be unnecessary after RP in low-risk patients even if adverse pathologic features are found. This additional treatment for low-risk patients could actually be overtreatment.

Multiple studies of patients with RP after AS versus those with immediate RP reported no increase in the rate of adverse pathologic features detected between the two groups [27, 28], and no significant difference in BCR-free survival [15], which indicates that AS does not affect patients. In addition, the fact that adverse pathologic features do not affect BCR-free survival, no matter the time point they are detected, suggests that adverse pathologic features in low-risk patients might not be associated with BCR. Adverse pathologic features in low-risk patients with RP could be clinically insignificant.

As the prevalence of IDCP increases, the risk of poor prognosis and BCR increases [11, 16]. However, much is still unknown about IDCP in RP specimens from low-risk patients. PCa with IDCP is more likely to have increased genomic instability, especially somatic MMR gene alterations. The NCCN guidelines state that patients with IDCP at biopsy should undergo germline testing but do not address what should be done if IDCP is found in the RP specimens [1]. Our findings suggest that IDCP in RP specimens from low-risk patients might not affect BCR. Further studies by risk stratification are needed, including investigation of the relations to gene mutations and prognosis.

This study had a few limitations. First, the study was retrospective, and the choice of RP depended on the opinion of the urologist and the patient’s compliance. Second, the overall risk analysis showed no significant difference in BCR-free survival between low-risk and intermediate-risk patients, owing to a small sample size of the low-risk patients in the overall risk analysis. To partially address this limitation, the number of patients included was increased by adding low-risk patients from the Japanese Red Cross Ise Hospital. However, the small sample size was a limitation of this study. Since our study is a short-term observation, additional long-term studies are required. Third, GG was upgraded in 76% of cases of low-risk patients at RP. Upgrading has been reported to occur in 36–71% of cases [29, 30], which in this study was slightly higher than existing reports. Insufficient sampling at the time of the biopsy could be the reason. Since biopsies are often performed at other hospitals, it is difficult to reexamine this issue. Fourth, cribriform carcinoma has a poor prognosis like IDCP, but it is difficult to truly distinguish IDCP from cribriform carcinoma. In this study, we diagnosed IDCP only when it could be reliably identified as IDCP by HE-stained slides alone.

## Conclusions

In our study, pathological prognostic factors did not affect BCR-free survival in low-risk patients according to the NCCN guidelines. Thus, additional treatment might be unnecessary even if pathological prognostic factors are observed in low-risk patients with RP.

## Data Availability

The datasets used and/or analyzed during the current study are available from the corresponding author on reasonable request.

## Abbreviations

PCa: Prostate cancer
GG: Grade group
PSA: Prostate-specific antigen
PSAD: PSA density
AS: Active surveillance
EBRT: External beam radiotherapy
BT: Brachytherapy
RP: Radical prostatectomy
BCR: Biochemical recurrence
LVI: Lymphovascular invasion
EPE: Extraprostatic extension
SM: Surgical margins
SVI: Seminal vesicle invasion
pT: Pathological assessment of the primary tumor
IDCP: Intraductal carcinoma of the prostate
